# High Concentrations of Se Inhibited the Growth of Rice Seedlings

**DOI:** 10.3390/plants13111580

**Published:** 2024-06-06

**Authors:** Ying Liu, Jiayu Ma, Feng Li, Xiang Zeng, Zhengwei Wu, Yongxiang Huang, Yingbin Xue, Yanyan Wang

**Affiliations:** Department of Agronomy, College of Coastal Agricultural Sciences, Guangdong Ocean University, Zhanjiang 524088, China; liuying85168@gdou.edu.cn (Y.L.);

**Keywords:** rice, selenium toxicity stress, physiological response, roots and leaves

## Abstract

Selenium (Se) is crucial for both plants and humans, with plants acting as the main source for human Se intake. In plants, moderate Se enhances growth and increases stress resistance, whereas excessive Se leads to toxicity. The physiological mechanisms by which Se influences rice seedlings’ growth are poorly understood and require additional research. In order to study the effects of selenium stress on rice seedlings, plant phenotype analysis, root scanning, metal ion content determination, physiological response index determination, hormone level determination, quantitative PCR (qPCR), and other methods were used. Our findings indicated that sodium selenite had dual effects on rice seedling growth under hydroponic conditions. At low concentrations, Se treatment promotes rice seedling growth by enhancing biomass, root length, and antioxidant capacity. Conversely, high concentrations of sodium selenite impair and damage rice, as evidenced by leaf yellowing, reduced chlorophyll content, decreased biomass, and stunted growth. Elevated Se levels also significantly affect antioxidase activities and the levels of proline, malondialdehyde, metal ions, and various phytohormones and selenium metabolism, ion transport, and antioxidant genes in rice. The adverse effects of high Se concentrations may directly disrupt protein synthesis or indirectly induce oxidative stress by altering the absorption and synthesis of other compounds. This study aims to elucidate the physiological responses of rice to Se toxicity stress and lay the groundwork for the development of Se-enriched rice varieties.

## 1. Introduction

Se (Selenium) is a critical element for organic life [1]. It is essential for various life forms, including humans, as it is a component of the amino acid selenocysteine (SeCys) [1]. Se is important for human and animal health [2]. It plays a vital role in maintaining physical well-being [3], particularly in terms of its antioxidant [4], anticancer [5], cardiovascular disease prevention [6], immune improvement [7], and other good auxiliary effects. The recommended daily intake of Se for humans is 55 µg (MCGs) [8]. As a trace element, Se is crucial for numerous biological activities, such as thyroid hormone metabolism, antioxidant defense, immune system strengthening, and prevention of certain types of cancer [9]. Epidemiological studies have shown a negative correlation between Se intake and mortality from various cancers [10]. Se is primarily obtained from food sources and some Se supplements, including plants, animals, and certain microorganisms [11]. In the human body, Se is metabolized into 25 selenoproteins, each of which serves distinct biological functions, including antioxidant and anticancer effects, as well as improving fertility and reproduction [12]. Furthermore, Se has been found to be beneficial for crop growth and protection against specific biological and abiotic stresses when it is maintained at appropriate concentrations [13].

In soils, Se levels range from 0.01 to 2.0 milligrams per kilogram, with a mean value of 0.4 milligrams per kilogram, except in selenic soils, where the Se content is >10 mg/kg [14]. The difference between the nutritional dose of Se and the maximum safe intake is very narrow. The general intake for the human body is 50~250 μg/d, and high levels of Se have detrimental effects on biological organisms [15]. Chronic Se poisoning occurs when individuals consume excessive amounts of Se over a prolonged period, and common symptoms in humans include hair loss, nail damage, and rashes [1]. Furthermore, studies indicate that excessive Se may contribute to the development of type 2 diabetes [16,17] and severe bowel disease [18]. In plants, superfluous Se interferes with nutrient absorption, inhibits the formation of photosynthetic pigments, causes oxide damage, and induces genotoxic effects [19]. Additionally, elevated Se levels above this threshold impede plant growth and development, thus reducing grain yield [20].

Plants primarily assimilate and transfer Se through phosphate and sulfate transport proteins, which accumulate Se in their roots [19,21]. Se has been proven to play a role in the adjustments to stress response, respiration, photosynthesis, abiotic stress survivability, oxidation resistance, and remission of heavy metal stress [22,23]. Land plants predominantly take up Se through active transport processes, specifically in the form of selenates or selenites, which are primarily absorbed through sulfate carrier proteins [24,25]. Selenites have also been found to enter root cells via phosphate transporters [26]. Generally, young leaves have a greater Se concentration during seedling growth than older leaves [27,28]. Within plant cells, Se primarily accumulates in vacuoles [29] and can be excreted through sulfate transporters present in flat bodies [30].

In plants, Se assimilation results in the production of ROS (reactive oxygen species), which are quickly neutralized by antioxidant substances [31]. Nevertheless, when the ratio of Se assimilability is very high or Se poisoning is present, an imbalance between ROS and antioxidant substances occurs, leading to oxidizing stress that influences different physiological response processes. These processes include chlorophyll deletion, suppression of the tricarboxylic acid cycle (TCA), and decreasing photosynthetic efficiency [31]. It is interesting that some ROS have been found to be involved in regulating genetic expression and influencing Se absorption [31].

Plants employ efficient mechanisms for alleviating stress, such as oxidation resistance systems, to minimize Se toxicity. These systems effectively eliminate excess ROS under Se stress [19]. Optimum Se concentrations stimulate the biosynthesis of antioxidants, thus impacting plants’ responses to biotic and abiotic stresses [21].

Plant hormones have a vital role in sustaining optimum cell activity through the regulation of different signaling pathways [32]. They can be produced interiorly in response to special stimuli or applied externally to regulate the growth of plants [32]. Furthermore, the interaction between plant hormones and Se contributes to a range of effects, including the maintenance of a REDOX balance, nutrient absorption, changes in development patterns, and improved crop yield and quality [33,34]. High concentrations of Se, acting as a potent pro-oxidant, promote plant physiological deterioration. The degree of damage is species-specific and concentration-dependent [35]. However, at trace concentrations, Se acts as an antioxidant in several plants. It replenishes metabolic pools, triggers antioxidant mechanisms, enhances the accumulation of nonenzymatic antioxidants, and boosts the activity of antioxidant enzymes [35].

As a crucial cash crop, rice (*Oryza sativa*) is globally recognized for its high yield and extensive cultivation [36]. Moreover, rice serves as an important source of Se for human consumption, and the Se level in rice determines the Se nutritional status of the Chinese population [37,38]. Research has indicated that rice cultivated in high-Se soil accumulates more Se than rice grown in low-Se soil [39]. Although cereals produced from naturally Se-rich soil can meet the daily Se requirements of individuals, approximately 72% of soil in China lacks Se, resulting in insufficient Se content in the plants growing on it [40]. Consequently, the Se content in most rice falls below the required levels, failing to meet the daily Se requirements of individuals [41,42]. In light of this, studies have demonstrated that rice possesses a remarkable ability to enrich Se and serve as a crucial carrier for Se, with the application of Se fertilizer markedly bolstering the Se content in rice [43,44]. Nevertheless, research has also highlighted that low levels of exogenous Se can enhance plant development, whereas high levels can restrain it [25,45]. Several studies have shown that increased Se fertilizer application leads to increased Se content in rice grains [46,47,48]. Furthermore, researchers are eager to identify suitable rice varieties with robust Se-enrichment capabilities, thus enabling the production of safe Se-rich rice with lower Se supply levels [49].

Current studies on Se in rice predominantly focus on Se accumulation and distribution [46,47,48,49]. However, the physiological response mechanism of rice to excessive Se remains unclear [50,51], necessitating further investigation. Consequently, this study aimed to examine the growth phenotype and physiological characteristics of rice subjected to different concentrations of sodium selenite and to explore gene expression in rice. The ultimate goals are to increase our understanding of the molecular regulatory network of Se in rice, elucidate the physiological response mechanism of rice to excessive Se, and provide a foundation for the cultivation of high-quality rice enriched with Se in a targeted manner, thus yielding far-reaching implications.

## 2. Results

### 2.1. Influences of Various Concentrations of Se on Rice Development

**Figure 1:**
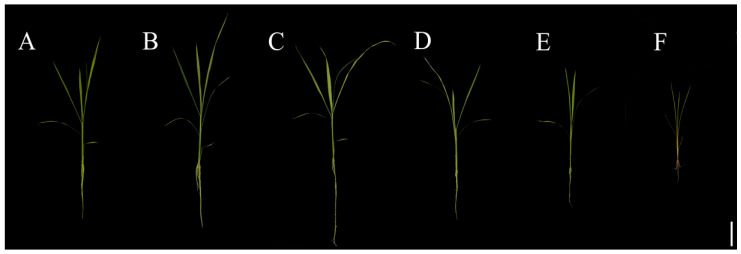

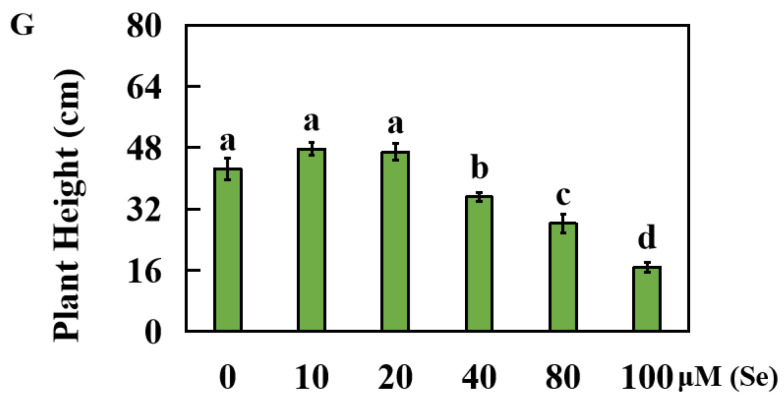
Phenotype of rice treated with different Se concentrations. Rice seedlings with a seedling age of 7 days were used for treatment with (**A**) 0; (**B**) 10; (**C**) 20; (**D**) 40; (**E**) 80; and (**F**) 100 μM Na_2_SeO_3_ for 20 days (bar = 5 cm). (**G**) The plant height of rice. The results are expressed as the mean and SD (standard deviation) from experiments repeated three times. Duncan’s multiple comparison method was used to compare the differences in Se toxicity among all the groups. Different lowercase letters marked on the column diagrams indicate that there were significant differences between these data (*p* < 0.05).

**Figure 2:**
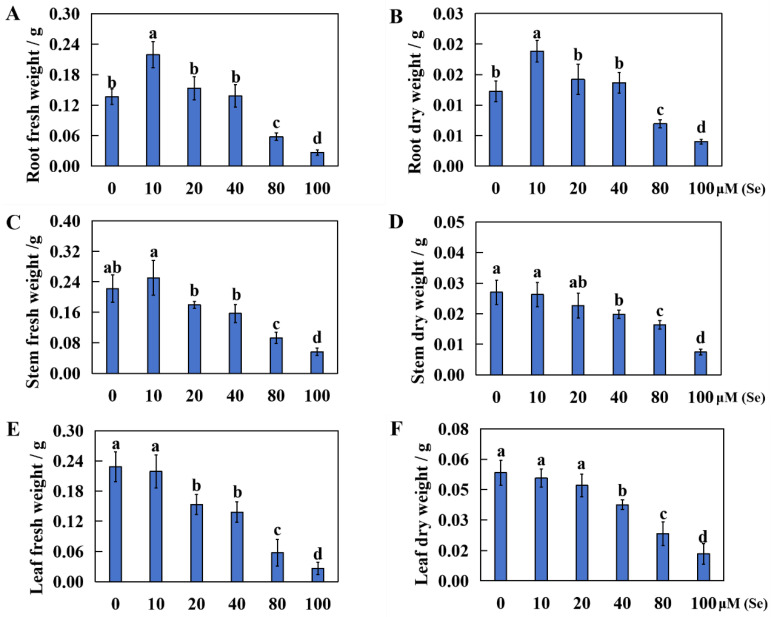
Results for various Se levels on the fresh and dry weight of rice roots, stems, and leaves. After treatment with 0, 10, 20, 40, 80, and 100 μM Se for 20 days, the dry and fresh weights were tested, and the CK was 0 μM Se. (**A**) Fresh weight of roots; (**B**) dry weight of roots; (**C**) fresh weight of stems; (**D**) dry weight of stems; (**E**) fresh weight of leaves; (**F**) dry weight of leaves. The results are expressed as the mean and SD (standard deviation) from experiments repeated three times. Duncan’s multiple comparison method was used to compare the differences in Se toxicity among all the groups. Different lowercase letters marked on the column diagrams indicate that there were significant differences between these data (*p* < 0.05).

To assess the effects of Se poisoning stress on rice seedling growth, we measured differences in the plant height and fresh and dry weight of rice plants treated with various levels of Se. The results showed that low Se concentrations promoted rice seedling growth, whereas high Se concentrations significantly impeded growth (Figure 1A–F). Compared with that of the control treatment (0 μM Se), the plant height of the rice plants treated with lower Se concentrations (10 and 20 μM) exhibited insignificant increases of 10.91% and 9.55%, respectively. However, higher Se levels (40, 80, and 100 μM) leaded to a notable reduction in plant height, with reductions of 17.20%, 33.55%, and 60.41%, respectively (Figure 1G). Taking 0 μM of Se treatment as the control (CK), the fresh root weight of rice increased significantly by 61.03% in the 10 μM Se treatment group, whereas there was no significant change in the 20 μM and 40 μM Se treatment groups. Higher concentrations of Se (80 and 100 μM) resulted in significant decreases of 58.09% and 80.15%, respectively, in the fresh root weight of the rice plants (Figure 2A). In terms of rice’s root dry weight, the 10 μM Se treatment group showed a significant increase of 54.79%, whereas there was no significant change in the 20 μM and 40 μM Se treatment groups. The rice’s root dry weights in the 80 μM and 100 μM Se treatment groups significantly decreased by 43.15% and 67.12%, respectively (Figure 2B).

Compared with that in the CK treatment group, the stem fresh weight in the 10 μM Se treatment group increased by 12.61%, but this increase was not statistically significant. The stem fresh weight in the 20 μM and 40 μM Se treatment groups did not significantly decrease. Nevertheless, higher levels of Se (80 and 100 μM) markedly decreased the fresh weight of the stems by 58.11% and 74.77%, respectively (Figure 2C). The stem weights in the 10 μM and 20 μM treatment groups did not significantly change, whereas those in the other treatment groups significantly decreased by 26.83%, 40.74%, and 72.59%, respectively (Figure 2D). Compared with that in the CK group, the rice leaves’ fresh weight from the 10 μM Se processing group did not significantly decrease, whereas there were significant decreases of 32.89%, 39.47%, 74.69%, and 88.59% in the other treatment groups (Figure 2E). The leaf dry weight did not significantly decrease in the 10 μM and 20 μM Se treatment groups compared to that in the CK group, whereas there were evident decreases of 29.88%, 56.59%, and 75.04%, respectively, in the other treatment groups (Figure 2F).

### 2.2. Influences of Various Levels of Se on Root Development in Rice

**Figure 3:**
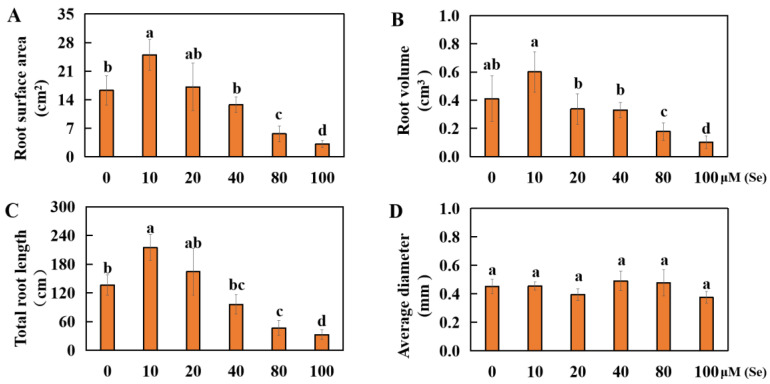
Results of rice root development under different selenium concentrations. (**A**) Surface area of roots; (**B**) volume of roots; (**C**) total root length; (**D**) mean diameter of roots. The results are expressed as the mean and SD (standard deviation) from experiments repeated three times. Duncan’s multiple comparison method was used to compare differences in Se toxicity among all the groups. Different lowercase letters marked on the column diagrams indicate that there were significant differences between these data (*p* < 0.05).

To analyze the effects of different Se concentrations on rice root development, changes in rice root growth were measured under varying Se concentrations. The CK group was treated with 0 μM of Se. The root surface area of the 10 μM treatment group significantly increased by 53.53% compared to that of the control group. However, there were no significant changes in the root surface area of the plants in the 20 μM and 40 μM Se treatment groups. Conversely, the root surface area in the other treatment groups decreased significantly, by 65.47% and 80.81%, respectively (Figure 3A). Similarly, the root volume in the 10 μM Se treatment group significantly increased by 46.06%, whereas that in the 20 μM and 40 μM Se treatment groups did not significantly change. The root volume in the other treatment groups decreased significantly, by 56.77% and 89.01%, respectively (Figure 3B). In terms of root length, a significant increase of 57.58% was observed under the 10 μM Se treatment. However, there were no significant changes in root length in the 20 μM Se or 40 μM Se treatment groups. On the other hand, the root length decreased significantly by 65.91% and 76.14%, respectively, in the other treatment groups (Figure 3C). The mean root diameter in each treatment group did not markedly change compared with that in the CK group (Figure 3D).

### 2.3. Effects of Se Stress on the Physiological Indicators of Rice

Under high Se stress, the physiological response indices of rice were significantly impacted. Moreover, the rice plants experienced pronounced growth stress under the Se treatment at concentrations of 80 and 100 μM. The severe dwarfing and yellowing observed were a direct result of the excessive growth stress caused by the 100 μM Se treatment. However, this concentration was deemed too high for research purposes, leading to the selection of 80 μM of Se as the stress concentration. A comparison between the control group treated with 0 μM of Se and the group treated with 80 μM of Se for 20 days revealed the significant influence of Se stress on the physiological indicators of roots, stems, and leaves in rice.

**Figure 4:**
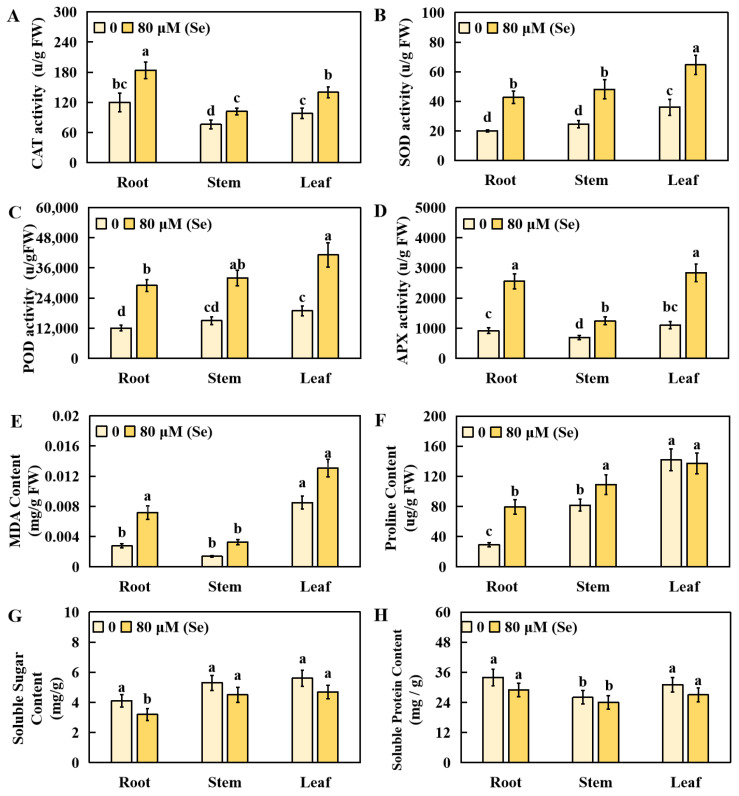
Effects of physiological indices in rice roots, stems, and leaves under selenium poisoning stress. The concentration of selenium toxicity was 80 μM Se, and the control group was 0 μM Se. Eight physiological indices were determined: the activities of (**A**) CAT (catalase), (**B**) SOD (superoxide dismutase), (**C**) POD (peroxidase), and (**D**) APX (ascorbate peroxidase) and the contents of (**E**) malondialdehyde (MDA), (**F**) proline, (**G**) soluble sugar, and (**H**) soluble protein. The results are expressed as the mean and SD (standard deviation) from experiments repeated three times. Duncan’s multiple comparison method was used to compare the differences in Se toxicity among all the groups. Different lowercase letters marked on the column diagrams indicate that there were significant differences between these data (*p* < 0.05).

Compared with those in the CK group, the CAT activity in the roots, stems, and leaves increased by 53.33%, 34.21%, and 42.86%, respectively, under Se stress (Figure 4A). Similarly, the superoxide (SOD) activity increased by 113.51%, 96.33%, and 79.44% in the roots, stems, and leaves, respectively, under high Se stress (Figure 4B). Under Se stress, the POD activity in the roots, stems, and leaves significantly increased by 139.67%, 113.34%, and 116.98%, respectively (Figure 4C). The APX activity also significantly increased by 178.26%, 78.99%, and 158.18% in the roots, stems, and leaves, respectively, under stress treatment (Figure 4D). The content of malondialdehyde in the roots, stems, and leaves significantly increased by 160.73%, 137.23%, and 54.29%, respectively, under Se treatment (Figure 4E). Compared with those in the CK group, the proline levels in the leaves did not significantly change under Se stress but increased significantly, by 172.50% and 33.58%, in the roots and stems, respectively (Figure 4F). Compared with those in the CK group, the soluble sugar levels in the leaves, roots, and stems under high Se stress tended to decrease overall, with a significant decrease of 21.95% in the roots (Figure 4G). The levels of soluble protein in the leaves, roots, and stems decreased under Se stress, but not significantly (Figure 4H).

### 2.4. Influences of Se Poisoning Stress on Chlorophyll Levels in Rice

**Figure 5:**
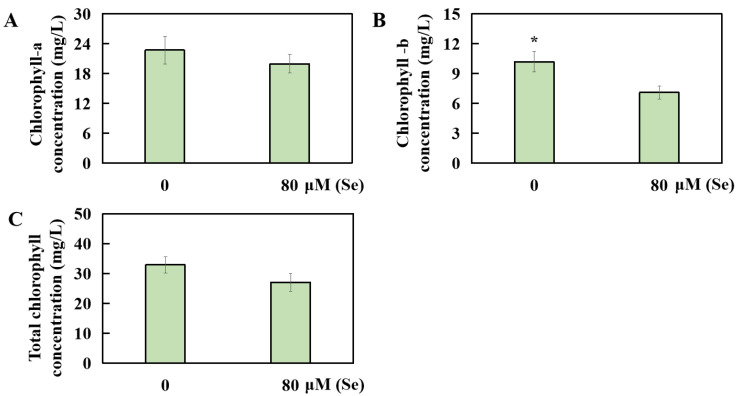
Changes in photosynthetic pigment content in rice leaves under different Se concentrations. The levels of (**A**) chlorophyll a, (**B**) chlorophyll b, and (**C**) total chlorophyll were measured. The results are expressed as the mean and SD (standard deviation) from experiments repeated three times. The *t*-test was used to compare the differences in Se toxicity among all the groups (* *p* < 0.05).

The Se stress treatment had an impact on the content of photosynthetic pigments in the rice leaves. Compared with that in the CK group treated with 0 μM of Se, the content of chlorophyll in rice decreased by 12.11% under the 80 μM Se treatment, although this change was not significant (Figure 5A). Conversely, the chlorophyll b content significantly decreased by 30.29% under the Se stress treatment (Figure 5B). The overall concentration of total chlorophyll decreased by 17.77%, but this change was not statistically significant (Figure 5C).

### 2.5. Influences of Se Stress on the Accumulation of Several Elements in Rice

The concentrations of Se, Zn, Cu, Fe, Mn, Ca, K, Al, Mg, and Na were determined after a 20-day treatment with 80 μM of Se. The samples were then dried until a constant weight was achieved. This research aimed to explore the influence of Se toxicity stress on the accumulation of these 10 elements in rice roots and leaves.

**Figure 6:**
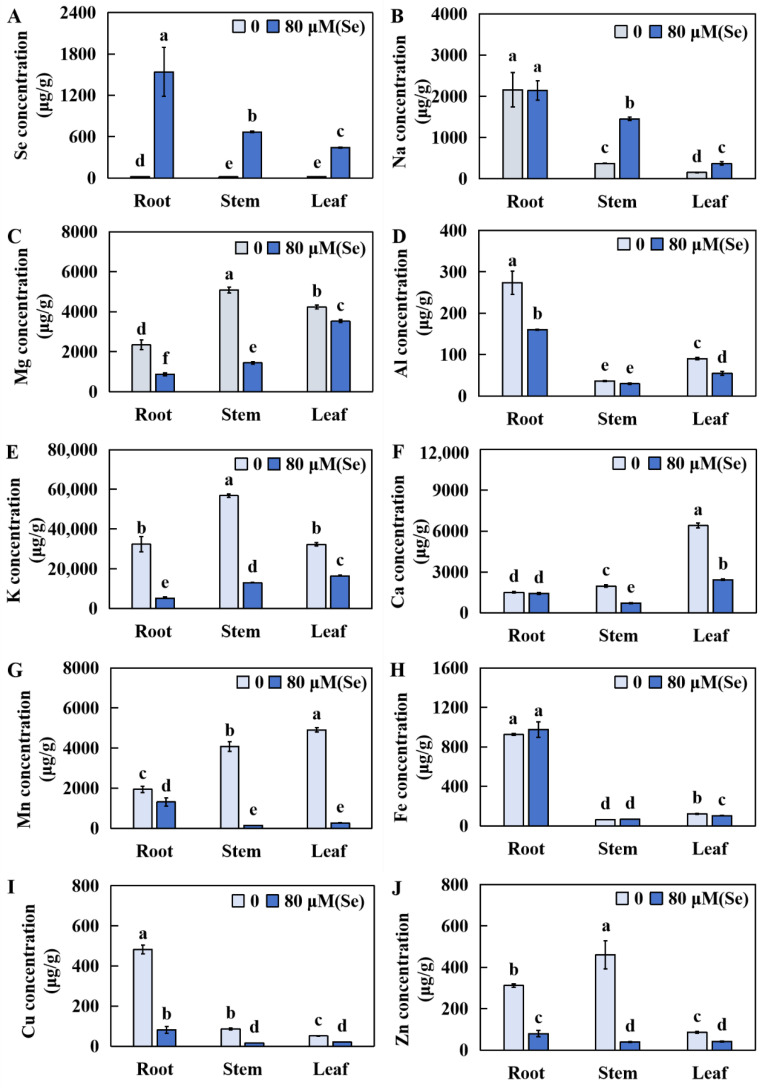
Accumulation of 10 elements in rice roots, stems, and leaves under Se toxicity stress. (**A**) Se (selenium); (**B**) Na (sodium); (**C**) Mg (magnesium); (**D**) Al (aluminum); (**E**) K (potassium); (**F**) Ca (calcium); (**G**) Mn (manganese); (**H**) Fe (iron); (**I**) Cu (copper); (**J**) Zn (zinc). The results were expressed as the mean and SD (standard deviation) from experiments repeated three times. Duncan’s multiple comparison method was used to compare differences in Se toxicity among all the groups. Different lowercase letters marked on the column diagrams indicate that there were significant differences between these data (*p* < 0.05).

In the Se stress treatment group, compared to those in the CK group, the Se concentrations in the roots, stems, and leaves significantly increased by 9003.18%, 14803.87%, and 4645.63%, respectively (Figure 6A). There was no obvious change in the Na levels in the roots, but a significant increase of 290.56% and 146.95% was detected in the stems and leaves, respectively (Figure 6B). The Mg levels in the roots, stems, and leaves decreased significantly by 62.77%, 71.69%, and 16.68%, respectively (Figure 6C). Although there was no obvious change in the Al levels in the rice stems, it significantly decreased in the roots and leaves by 41.49% and 38.95%, respectively (Figure 6D). The K levels in the roots, stems, and leaves decreased significantly, by 84.17%, 77.18%, and 49.39%, respectively (Figure 6E). The Ca contents in the roots did not significantly change, whereas the Ca contents in the stems and leaves significantly decreased by 64.09% and 62.23%, respectively (Figure 6F). The contents of Mn in the roots, stems, and leaves were markedly reduced by 32.37%, 96.68%, and 94.52%, respectively (Figure 6G). The Fe contents did not change significantly in the roots and stems but significantly decreased in the leaves by 15.92% (Figure 6H). The Cu levels in the roots, stems, and leaves decreased significantly, by 20.95%, 81.84%, and 59.19%, respectively (Figure 6I). Furthermore, the Zn contents in the roots, stems, and leaves decreased markedly, by 75.59%, 91.72%, and 51.52%, respectively (Figure 6J).

### 2.6. Determination of Five Kinds of Hormones from Roots and Leaves in Rice

**Figure 7:**
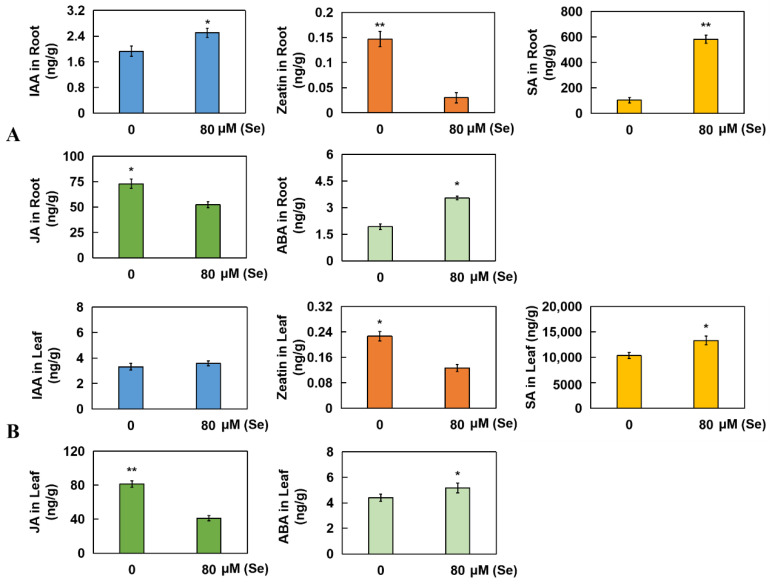
Changes in five hormone levels in rice roots and leaves under Se stress. The five hormones measured in rice roots (**A**) and leaves (**B**) were IAA, zeatin, SA, JA, and ABA. The results are expressed as the mean and SD (standard deviation) from experiments repeated three times. The *t*-test was used to compare differences in Se toxicity among all the groups (* *p* < 0.05, ** *p* < 0.01).

Under high Se stress, the contents of IAA (indoleacetic acid), SA (salicylic acid), and ABA (abscisic acid) in the roots significantly increased by 29.71%, 463.71%, and 83.91%, respectively. However, the levels of zeatin and JA (jasmonic acid) were significantly reduced by 89.59% and 28.38%, respectively (Figure 7A). Compared with that in the CK group, the level of IAA in the leaves did not significantly change under Se stress. However, the contents of zeatin and JA were significantly reduced by 44.12% and 49.69%, respectively, whereas the contents of SA and ABA were markedly increased by 28.38% and 17.62%, respectively (Figure 7B). The retention time and HPLC chromatograms of zeatin, SA, JA, IAA, and ABA are displayed in Figure S1.

### 2.7. Effects of Se Poisoning Stress on the Relative Expression of Nine Related Genes in Rice

**Figure 8:**
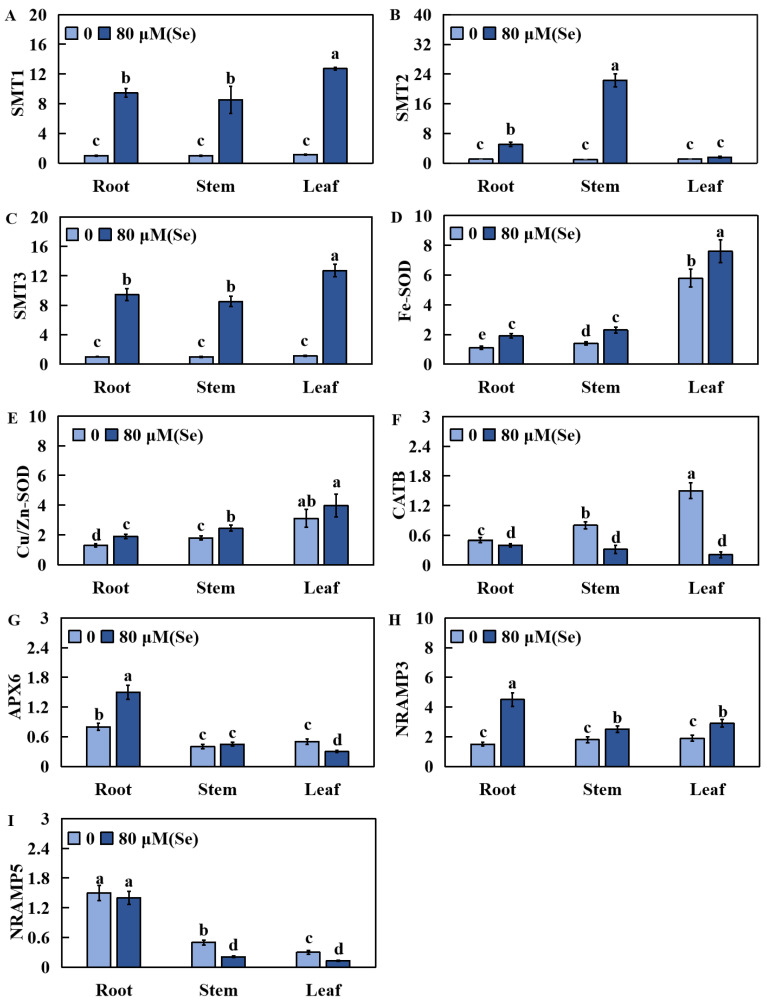
Relative transcript levels of 9 related genes in leaves, stems, and roots of rice under Se stress. (**A**) SMT1; (**B**) SMT2; (**C**) SMT3; (**D**) Fe-SOD; (**E**) Cu/Zn-SOD; (**F**) CATB; (**G**) APX6; (**H**) NRAMP3; (**I**) NRAMP5. The results are expressed as the mean and SD (standard deviation) from experiments repeated three times. Duncan’s multiple comparison method was used to compare differences in Se toxicity among all groups. Different lowercase letters marked on the column diagrams indicate that there were significant differences between these data (*p* < 0.05).

To investigate the effect of selenium stress on the expression of nine related genes in leaves, the relative transcript levels of three types of genes were measured via real-time fluorescence. These gene categories included genes related to selenium metabolism (*SMT1*, *SMT2*, and *SMT3*), physiological response genes (*Fe-SOD*, *Cu/Zn-SOD*, *CATB*, and *APX6*), and ion transport genes (*NRAMP3* and *NRAMP5*). *SMT1*, *SMT3*, *Fe-SOD*, *Cu/Zn-SOD*, *APX6*, and *NRAMP3* were upregulated in the roots (Figure 8A,C–E,G,H). *CATB*, on the other hand, was significantly downregulated in the roots (Figure 8F), whereas *SMT2* and *NRAMP5* did not significantly change in the roots (Figure 8B,I). In the stem, the significantly upregulated genes were *SMT1*, *SMT2*, *SMT3*, *Fe-SOD*, *Cu/Zn-SOD*, and *NRAMP3* (Figure 8A–E,H). *CATB* and *NRAMP5*, on the other hand, were significantly downregulated in the stem (Figure 8F,I), whereas *APX6* did not significantly change (Figure 8G). In the leaves, *SMT1*, *SMT3*, *Fe-SOD*, *APX6*, and *NRAMP3* were significantly upregulated (Figure 8A,C,D,G,H). Conversely, *CATB* and *NRAMP5* were significantly downregulated (Figure 8F,I), whereas *SMT2* and *Cu/Zn-SOD* did not significantly change (Figure 8B,E).

## 3. Discussion

The concentration and availability of Se in crops are affected by soil characteristics and environmental factors [14]. Suitable concentrations of Se can enhance plant development and improve stress resistance, but excess Se can be toxic [14]. Hamilton identified three levels of biological activity for Se. The first level suggests that low Se concentration doses can facilitate plant development, whereas the second level suggests that moderate doses can maintain homeostasis processes. The third level suggests that high doses of Se can have harmful consequences [52,53]. For example, low Se concentrations significantly promote the growth of apple (*Malus pumila*) plants, but once the Se concentration exceeds 12 μM, plant growth is notably inhibited, particularly in the roots [54]. High concentrations of Se in plants can induce phytotoxicity by interfering with metabolism, resulting in chlorosis, cell membrane degradation, senescence, and reduced grain yield [55]. Studies have shown that sodium selenate has dual effects on rice seedling growth and development: low levels facilitate development, whereas high levels restrain development [56]. Similarly, the results of this study demonstrated that Se has a similar effect on rice. Under low Se treatment, rice seedling growth increased, but under high Se treatment, the plant height, root length, root volume, total number of roots, root surface area, and dry and fresh weights of the rice significantly decreased due to high Se stress. Consequently, these changes result in stunted plants, yellow leaves, and hindered growth. These effects are likely due to the strong binding of Se to the cell wall in rice root cells. An excessive Se concentration disrupts the structure and stability of rice root cells, leading to cell deformation, cell death, and diminished nutrient absorption capacity in rice plants.

In addition to its significant effect on human health, Se also has a crucial role in the antioxidant protection of plants [56]. Se has the ability to stimulate growth, resist pathogens, and confer tolerance to oxidative stress [57]. When plants suffer from stress conditions, they create excess ROS, which can result in damage to cell components, the destruction of cell membranes, and the inhibition of plant growth [58]. To combat oxidative stress, plants rely on their antioxidant resistance system and osmotic adjustment system, both of which contribute to their resistance to such stress [59].

When Se is used in conjunction with other nutrients during fertilization, it can activate enzymes such as SOD and catalase (CAT) depending on the dosage. These enzymes are essential for reducing oxidative stress in plant cells and are especially active in the presence of Se [60]. Se in plants can function as a scavenger of ROS at low concentrations by increasing antioxidant metabolism, but at higher concentrations, it can actually enhance oxidative stress [61]. SOD, which is the main antioxidant enzyme responsible for scavenging ROS, helps maintain the balance of reactive oxygen metabolism and protect the integrity of the cell membrane structure [62].

To counter excessive Se accumulation, plants have developed an antioxidant system regulated by enzymes such as SOD, CAT, APX, glutathione reductase (GR), and glutathione (GSH). The rice plants treated with Se exhibited increased SOD and CAT activity, indicating the importance of these antioxidant enzymes [61]. Consequently, it is crucial to measure the antioxidase activities and osmoregulatory components to assess the metabolic activity and overall health of cells [61].

It is widely recognized that Se can increase SOD activity and gene expression levels [61]. In a previous research report, SOD activity was increased by adding 0.5 mg/kg of Se to rice [61]. The control of SOD activity in response to Se is influenced by three main factors in plants, as previously reported [45,57]. The first factor is the stress level [45,57]. Under low stress conditions, the antioxidant capacity of plants is sufficient, whereas under high stress conditions, it may be necessary to increase SOD activity, which can be accomplished by adding Se [45,57]. The second element affecting the activity of SOD is the concentration of Se [45,57]. The third factor is connected with the concentration of SOD helper factors, such as Zn, Cu, Mn, and Fe [45,57]. SOD has an important role as the first defense against antioxidant systems in plants [62]. The family of plant SOD genes can be grouped into three categories on account of their metal helper factors: FeSOD, Cu/ZnSOD, and MnSOD [62]. In oilseed rape (*Brassica napus*), various genes, including *BnFeSOD4*, *BnFeSOD5*, *BnFeSOD6*, *BnMnSOD2*, *BnMnSOD10*, *BnCuSOD1*, *BnCuSOD3*, and *BnCuSOD14*, were markedly upregulated when it was subjected to different abiotic stresses, such as drought, salt, cold, and flooding [63]. In this study, the expression of SOD in the stems, leaves, and roots of rice subjected to Se poisoning stress significantly increased, whereas the levels of Cu and Zn significantly decreased, consistent with previous research results. This suggests that the absorption of Fe, Cu, and Zn in rice might be altered under the influence of Se, which could affect the differential expression of *Cu/ZnSOD* and *FeSOD* in rice as well as the SOD activity in rice leaves and roots. The family of POD genes plays a part in the biosynthesis of oxidoreductases in grapes (*Vitis vinifera*) [64]. The class III plant POD is a plant-specific oxidant and is widely distributed in animals, plants, and microorganisms [65]. Previous studies have demonstrated the involvement of PODs in various processes, such as lignification, plant defense, development, and germination, as well as their mechanisms of action, including substrate oxidation, reactive oxygen species regulation, and free radical formation, particularly in lignification [66]. For instance, PODs (AtPrx72 and PtrPO21) in *Arabidopsis thaliana* and Populus trichocarpa play a crucial role in leaf lignification [67]. The overexpression of *POD* in *A. thaliana* (*AtPrx22*, *AtPrx39*, and *AtPrx69*) has been shown to improve cold tolerance [68]. In this study, Se poisoning induced differential *POD expression*, leading to a significant increase in *POD* expression. These changes could be attributed to the irreversible damage caused by Se to the formation of xylem in leaves, triggering defense stress in rice plants, which affects the capacity of resistance to oxidation in rice as well as the development of leaves and roots. CAT is primarily found in peroxisomes and catalyzes the conversion of H_2_O_2_ to water [69]. The expression level of *CAT* in some crops is affected by phytohormones, such as ABA, which induces *AtCAT1* expression in *A. thaliana* [69]. CATB, also known as catalase isoenzyme B, works together with APX to clear H_2_O_2_ and regulate the redox balance. Specifically, CTAB interacts with OsAPX5 and OsAPX6, which are located in the mitochondria [70]. In oilseed rape, various genes, including *BnCAT13*, *BnCAT11*, *BnCAT3*, and *BnCAT1*, were markedly upregulated when it suffered from GA, ABA, salt, and low-temperature treatments [71]. Previous research reports have shown that genes such as *AhAPX4*, *AhAPX17*, and *AhAPX19* in peanuts (*Arachis hypogaea*) were significantly upregulated under abiotic stress and hormone treatments (SA and ABA), suggesting that the synthesis of APX is stimulated by various environmental stresses and that the differential expression of *APX* is related to hormone concentrations in peanut cells [72]. Therefore, the variations observed in APX and CAT activities in the current study might be attributed to the oxidative damage induced by Se stress as well as changes in hormone concentrations in rice cells.

In the current study, compared with those in the control group, the enzymatic activities of APX, CAT, POD, and SOD in the stems, leaves, and roots of the rice plants under Se stress were significantly elevated. The increase in these antioxidase activities suggested that the cell membrane system of the rice was partially damaged by Se poisoning. CAT, APX, SOD, and POD have crucial roles in preserving plant cell membrane systems during Se poisoning [73]. Similar physiological indicators have been surveyed when subjected to other types of abiotic stresses, such as in aluminum-treated soybeans (*Glycine max*) [74].

Furthermore, soluble sugar, proline, and soluble protein contribute to mitigating the effects of high Se stress [75]. This study revealed changes in soluble protein, proline, and soluble sugar levels. Under high Se stress, the soluble protein and soluble sugar levels decreased to varying degrees in the stems, leaves, and roots of rice, whereas the proline content increased. These findings suggest that rice responds collaboratively to Se poisoning by regulating the levels of osmoregulatory constituents such as proline, soluble sugar, and soluble protein. MDA is a final product of unsaturated fatty acid overoxidation, which can injure cell membranes when it accumulates [76]. The MDA content serves as an important indicator of lipid overoxidation in plants, and lipid peroxidation and protein changes induced by ROS are also controlled by high Se concentrations in plants [61]. In this study, the MDA content significantly increased in the stems, leaves, and roots of the plants that suffered high Se stress. This increase may be attributed to the elevated Se concentration and the additional peroxidation damage caused by Se poisoning, resulting in a progressive increase in malondialdehyde level.

In summary, under high Se stress, the oxidation resistance protection system and osmotic regulation system in rice might have important functions, resulting in changes in the levels of APX, POD, SOD, CAT, soluble protein, proline, MDA, and soluble sugars. These changes might serve to protect rice cells and mitigate oxidative damage.

There was a significant increase in the accumulation of Se in the rice stems, leaves, and roots after Se treatment compared with that in the control group. This augmentation might be the leading reason for Se toxicity in rice plants. Previous research reports have demonstrated that the application of selenocysteine to roots leads to a notable increase in extractable Se content in rice leaves [51]. Lin et al. reported that the addition of selenate to soil is the most efficient solution to enhance the Se content in rice grains [77]. It is important to note that in this research, the contents of Mg and K in the rice leaves, stems, and roots decreased distinctly. Past research has shown a synergistic association between Mg and K in plants and that K and Mg have synergistic effects on processes such as photosynthesis, carbohydrate transport and distribution, nitrogen metabolism, and the regulation of expansion [78]. The interaction between Mg and K occurs not only during the root absorption process but also during root-to-bud translocation, distribution, and utilization [79]. Similarly, the findings suggest that Mn plays a synergistic role in the accumulation of Al [80]. Ca is a component of cell wall pectin and affects the mobility of cytomembranes in plants [81]. In the current study, the decrease in the Ca, K, Mg, and Al contents in the rice under Se toxicity stress might have been due to the high level of Se taking up a large quantity of combining sites in the rice cells.

Selenocysteine methyltransferase (SMT) is a crucial enzyme in the plant Se metabolism pathway [82]. Studies on transgenic *Arabidopsis thaliana* plants that overexpress *SMT* have indicated that SMT has a function in the synthesis of MeSeCys and MeCys, suggesting that SMT may catalyze the methylation of selenocysteine and cysteine [82]. LeDuc et al. successfully cloned the SMT gene from Se-enriched Indian mustard (*Brassica juncea*) plants, and the resulting transgenic plants exhibited improved tolerance and accumulation of selenite [83]. These findings imply that SMT has significant potential for regulating the Se tolerance and Se enrichment ability of crops [84]. The results revealed that the SMT-related genes *SMT1*, *SMT2*, and *SMT3* were significantly upregulated under Se toxicity stress in this study. This could be attributed to the application of exogenous Se promoting the expression level of genes connected with the Se metabolism pathway in rice, thus facilitating the absorption of large quantities of Se by individual plants.

The family of NRAMPs is crucial for the uptake and transport of metals in crops, with peanut (*Arachis hypogaea*) *AhNramps* expressed mainly in stamens, immature seeds, and roots [85]. The NRAMPs play a vital role in transporting metallic ions through cytomembranes [86]. For example, the expression levels of *GmNRAMP5* and *GmNRAMP1* in soybeans are upregulated under Cu toxicity stress [86]. Both OsNRAMP3 and OsNRAMP5 are located in the plasma membrane and contribute to the absorption or transport of Mn, with the latter also involved in Fe absorption and transport [87,88]. In this study, *OsNramp5* and *OsNramp3*, members of the rice NRAMP family, exhibited differential expression in response to Se toxicity. These findings suggest that the NRAMP family in rice might have an important function in the resistance of rice to Se poisoning and could impact the absorption or transport of certain ions in rice.

In recent years, several studies have reported the influence of ABA on Se and heavy metal uptake and accumulation in plants [89,90,91]. ABA is a crucial hormone for plant resistance to metal ion toxicity [92]. Furthermore, ABA primarily plays functions in the elongation and meristem regions of plant roots [92]. In addition, ABA adjusts osmotic pressure equilibrium and stomatal opening and closing, thereby enhancing tolerance to adversity [93]. Similarly, the foliar application of ABA under Se toxicity stress has been found to stimulate the production of stress response phytohormones (such as SA (salicylic acid) and JA (jasmonic acid)), ultimately improving the Se resistance of plants [94]. In the current research, the ABA content in the rice roots markedly increased when suffering from Se poisoning, potentially contributing to Se poisoning in rice. Furthermore, the results might be due to Se-induced genes related to ABA synthesis in rice roots, indicating that increasing the ABA content can enhance the resistance of rice plants to Se toxicity stress.

SA promotes plant stress resistance by regulating photosynthesis and proline and nitrogen metabolism and stimulating the oxidation resistance system [95]. SA has been proven to decrease K outflow and Na afflux, thereby maintain ion balance and saline–alkaline resistance [95]. Adding Se to tomato (*Solanum lycopersicum*) roots and leaves under saline–alkali stress has been found to activate the SA biosynthesis pathway and increase SA levels, consequently improving tomatoes’ saline–alkaline resistance [53]. Since both SA and Se facilitate oxidation resistance systems, they may have synergistic effects through feedback loops [53]. SA participates in adjusting various facets of healthy plant development, including protein biosynthesis, signal transduction, material transportation, and so on [96]. Some studies have shown that SA can alleviate selenium toxicity in rice [97]. SA can maintain selenium homeostasis and reduce the oxidative stress induced by selenium stress by increasing the level of ascorbate and the activities of antioxidant enzymes such as superoxide dismutase, catalase, glutathione peroxidase, and glutathione reductase [97]. In addition, SA protects rice from the harmful effects of methylglyoxal by stimulating the glyoxalase activity [97]. In the present research, the SA levels in the rice leaves and roots markedly increased when suffering from Se poisoning. This could be due to Se-induced SA synthesis, leading to elevated SA levels in rice roots and leaves. Consequently, the phytohormone distribution and level in the rice leaves and roots were influenced, resulting in poor rice seedling growth.

JA acquires both biological and abiotic stress survivability by stimulating the oxidation resistance system and interacting with other phytohormones [98]. JA is related to Se tolerance, as Se addition activates JA synthesis and signal transduction, thus maintaining a high antioxidant content in Se superaccumulators [99]. During pathogenic microorganism infections, JA activates a signal pathway that triggers plant self-protection responses, including the synthesis and biological accumulation of plant antitoxins and pathogenic microorganism growth suppressor substances. It also alters the cytoderm structure to inhibit pathogenic microorganism entry [100]. Analogously, Se enhances the biosynthesis of antioxidant substances that can eliminate stress products, thereby improving disease enduring [99]. Jasmonate compounds include JA and its derivative methyl jasmonic acid (MeJA), which are growth regulators and signaling molecules associated with plant damage and can regulate plant growth and stress [101]. In addition, a low concentration of MeJA (0.1–1.0 μM) inhibited the non-reversible toxicity of rice seedlings induced by high selenium by enhancing the antioxidant system, reducing the content of H_2_O_2_ and MDA, and thereby alleviating the toxicity of high selenium treatment on rice seedlings [101]. Under Se poisoning, the level of JA in the rice roots and leaves significantly decreased. This decrease might be due to an antagonistic interaction between SA and JA, as an increase in the SA level led to a decrease in the JA level.

The stabilization of zeatin contents reflects plants’ coping styles in response to environmental changes [102]. Zeatin is primarily created by cells that quickly divide, such as in buds or root tips, and has a crucial role in enhancing root elongation, leaf expansion, the germination of seeds, and cell division and differentiation in plants [103]. Studies have revealed that under NaCl stress treatment, the zeatin concentration in tomato leaves (*Solanum lycopersicum*) decreases significantly within 24–72 h after treatment. Tomatoes respond to this stress by adjusting their zeatin concentration [104]. The zeatin content also significantly reduced the level of suffering from Se poisoning. This decrease might be due to the induction of Se, which affects the expression level of related genes and hinders zeatin biosynthesis. Alternatively, this may be caused by damage to hormone transport in roots due to Se toxicity stress, which inhibits the growth of rice roots.

IAA is a crucial plant hormone essential for various aspects of plant growth and development. It is the most extensively studied auxin in plants [105]. IAA exerts pleiotropic effects in nearly every step of plants’ life cycles, such as cell division and differentiation, plant organogenesis, cell development, physiological response to stress, and so on [106]. The metabolic activity and polar apportion of IAA are affected by metallic ions [107]. The results from studies have indicated that IAA has a vital impact on the physiological and biochemical processes of higher plants, regulating plant growth, development, and stress response [108]. In this research, the concentrations of IAA in the rice roots markedly increased when they were subjected to Se poisoning, indicating that Se stress inhibits the growth of rice roots. This increase in IAA may be due to high Se stress triggering the defense mechanism of roots, resulting in substantial production of IAA in the roots to cope with adversity.

**Figure 9:**
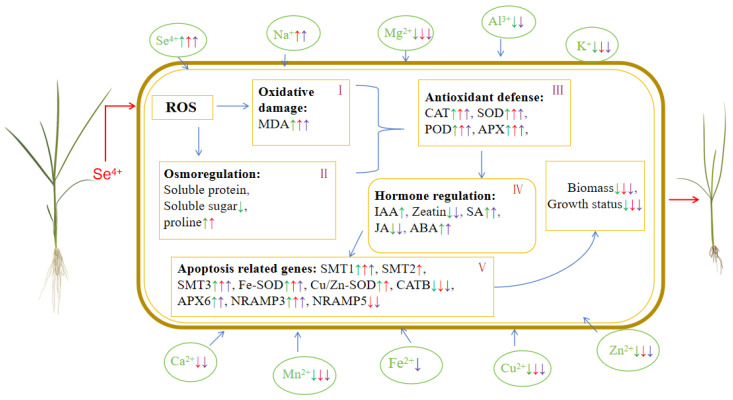
Regulatory pathways of high Se stress in rice. The rice plants on the left are the control group, and the rice plants treated with Se stress are on the right. (**I**) Oxidative damage; (**II**) osmotic adjustment; (**III**) antioxidant defense; (**IV**) hormone regulation; (**V**) expression of related genes. Green arrows indicate significant changes in physiological response indicators or gene expression levels in roots, red arrows indicate significant changes in stems, and purple arrows indicate significant changes in leaves.

Based on the current results of the present study, the physiological regulation mechanisms of rice plants in response to Se poisoning stress were summarized in Figure 9. Firstly, when suffering from Se toxicity stress, the uptake of selenium via rice root cells significantly increased. Under stress conditions, plants generate a large number of reactive oxygen species to cope with stress. Excess Se led to intracellular oxidative damage, and the MDA content served as an important indicator of lipid peroxidation in the cytomembranes of the plants, leading to an increase in the MDA level in various parts of the rice plants (Figure 9I). The rice cells regulated their milieu interne by means of osmotic adjustment substances to maintain an intracellular steady state, as evidenced by the changes in the concentrations of proline, soluble protein, and soluble sugar (Figure 9II). Subsequently, due to the influx of large amounts of Se, the rice plants developed an antioxidant system to balance the increasing amount of ROS attributed to excessive Se accumulation. This led to an improvement in the enzymatic activity of antioxidases (POD, SOD, APX, and CAT) (Figure 9III). Under Se stress, the rice plants mitigated stress by modulating their own hormone levels, resulting in significant variations in the concentrations of phytohormones (JA, SA, Zeatin, IAA, and ABA) in their leaves and roots (Figure 9IV). The changes in the growth of the rice plants under Se stress were jointly determined by genes related to Se metabolism, antioxidase activity, and ion transport (Figure 9V). Consequently, the weight of the rice stems, leaves, and roots decreased, and plant growth was inhibited.

## 4. Materials and Methods

### 4.1. Plant Materials

The rice variety used in the present study was Haihong 11 (Se sensitivity), which was generously donated by Professor Zhou Hongkai and cultivated at the plantation base of the Coastal Agricultural Sciences College of Guangdong Ocean University (East longitude: 110.30311, Northern latitude: 21.15005).

### 4.2. Material Handling

Rice seeds with full grains were chosen. The rice seeds were rinsed with tap water and then disinfected with 1.5% sodium hypochlorite (Solarbio, Beijing, China) for 30 min. After the seeds were thoroughly washed with deionized water 5 times, they were placed on moist absorbent paper in a glass culture dish (Devan, Wuxi, Jiangsu, China) with a diameter of 13.5 cm for 7 days of germination culture. After that, the rice seedlings were transplanted to a plastic bucket (5 L) with a 4 L hydroponic nutrient solution for 10 days of strong seedling culture. Finally, rice seedlings were treated by adding 0, 10, 20, 40, 80, or 100 μM sodium selenite (Na_2_SeO_3_) [50,109,110] (Solarbio, Beijing, China) and keeping them in a hydroponic nutrient solution for 20 days. The rice seedlings were cultivated in a growth chamber at 24–29 °C (daytime)/19–23 °C (night), with a light and dark cycle of 12/12 h, a relative humidity of 70%, a light intensity of 2000 lux, and Kimura Culture B nutrient solution (Table S1) [111]. Then, 8–10 rice seedlings were treated with each concentration. The experiment was repeated three times for each concentration treatment.

### 4.3. Determination of Fresh and Dry Weight and Plant Height of Rice

The fresh weight and plant height of the rice were measured immediately after harvest. Fresh rice seedlings were accurately weighed using electronic balances (Sartorius, Gottingen, Germany). Prior to dry weight measurement, the samples were moved into a constant-temperature drying oven (Yiheng, Shanghai, China) at 80 °C for 7 days [112].

### 4.4. Surveying of Root Phenotype Data

A Win RHIZO LA6400XL root scanning system (Regent, Vancouver, BC, Canada) was utilized to scan the root phenotype data of the rice plants, and images of the roots of each individual plant were obtained. Subsequently, Win RHIZO (WinRHIZO 2013e Professional Edition, Regent, Vancouver, BC, Canada) software was adopted to survey root surface area, total root length, number of root tips, and root volume. The measurements were repeated four times [113].

### 4.5. Surveying of Metal Ion Levels in Rice Stems, Leaves, and Roots

The metal ion levels in the stems, leaves, and roots of rice treated with 0 or 80 µM Se were assessed. Approximately 0.2 g of biologically dry sample was moved into a polytetrafluoroethylene crucible (Yaron Instrument, Shijiazhuang, China), wetted with a little water, and supplemented with 5 mL of 8 M HNO_3_ (Solarbio, Beijing, China). The mixture was placed at room temperature for 1 day until the dry sample was fully dissolved in nitric acid. Subsequently, the mixture was transferred to an electric heating plate (DB-XWJ, MITR, Changsha, China) and gradually heated to 180 °C for digestion. Upon reaching a minimal liquid state in the crucible, the crucible was decanted. After slight cooling, 2 mL of 8 M HNO_3_ and 2 mL of 30% H_2_O_2_ (Solarbio, Beijing, China) were supplemented, and the mixture was digested on an electric heating plate (Binzhenghong GS-type, Nanjing, China) at 180 °C. Finally, the mixture was heated and evaporated until nearly dry, after which 1 mL of concentrated nitric acid was added. After slight heating and leaching, all the extract and residue were transferred into 25 mL colorimetric tubes (Tianbo borosilicate glass, Tianjin, China) with distilled water and brought to a constant volume. The contents of Se, Zn, Fe, Ca, Mn, K, Cu, Al, Mg, and Na in the liquid treated with 0 and 80 μM Se were tested by ICP–AES (inductively coupled plasma atomic emission spectrometry) (Hitachi PS7800, Tokyo, Japan). Each index was measured four times [114].

### 4.6. Determination of Physiological Response Indices and Chlorophyll Content in Rice

The physiological response indices and chlorophyll content of rice were determined as follows: The physiological indices of rice roots subjected to two different concentrations of Se (0 and 80 μM) were assessed. Physiological data, including data on enzymatic antioxidant systems (SOD, CAT, POD, APX activity) and osmoregulatory substances (MDA, soluble protein, soluble sugar, proline), were analyzed. Additionally, the chlorophyll content (chlorophyll a, chlorophyll b, and total chlorophyll) in the rice leaves was measured following Se stress treatment. The chlorophyll measurements were conducted as follows: 0.2 g of fresh leaves was extracted from each treated plant, minced with scissors, and soaked in a 95% ethanol (Solarbio, Beijing, China) solution for 12 h in the absence of light. Subsequently, an ultraviolet spectrophotometer (Yuexi UV5100B, Shanghai, China) was utilized to survey the absorbance at 663 nm, 645 nm, and 652 nm, and the OD value was recorded [115]. The content of soluble protein was tested using the Coomassie brilliant blue method [116]. The level of soluble sugar was surveyed using the anthrone method [116]. The proline level was surveyed by extracting sulfosalicylic acid (Solarbio, Beijing, China) and subsequently measuring its content via the ninhydrin reaction [117]. The MDA content was determined using the TBA method (thiobarbituric acid) [118]. The activity of POD was surveyed using the guaiacol method [119]. The activity of SOD was determined using the nitroblue tetrazolium (NBT) method [120]. CAT activity was measured using a spectrophotometer [121]. The APX activity was surveyed using a spectrophotometer at 290 nm with ascorbic acid (Solarbio, Beijing, China) and a hydrogen peroxide solution (Solarbio, Beijing, China) [122].

### 4.7. Determination of the Levels of Six Kinds of Hormones in Rice Leaves and Roots

The levels of six hormones in rice leaves and roots treated with 0 and 80 μM Se were determined. Following the reported method [112], the operating solution was prepared, and the hormone standard curve was calculated to extract the hormones from the rice roots and leaves. For this experiment, an HPLC (high-performance liquid chromatography) system (Agilent 1290, Santa Clara, CA, USA) in conjunction with an AB Qtrap6500 mass spectrometer (AB, Madison, WI, USA) was used. The mobile phase was pumped into the system by an HPLC binary pump and mixed, and the sample to be tested was injected into the mobile phase using an automatic sampler before entering the chromatographic column. The components of the sample were separated based on their retention time in the chromatographic column and then entered the ion source sequentially. The levels of the endogenous hormones IAA, ABA, JA, SA, and zeatin in rice roots and leaves were detected using multiple response detection (MRM) mode scanning [123]. The Qtrap6500 mass analyzer consisted of Q1, Q2, and Q3 triple mass spectrometry in series, where Q1 separated the molecular ions based on the mass–charge ratio (*m*/*z*), Q2 served as a collision chamber to further split the parent ions into fragment ions, and Q3 functioned as a four-stage bar and a linear ion trap. Internal standard substances, including IAA, ABA, JA, SA, zeatin, and GA_3_ (Sigma–Aldrich, St. Louis, MO, USA), were used. External standard substances such as deuterated abscisic acid (D-ABA), deuterated gibberellin (D-GA4), deuterated indole acetic acid (D-IAA), deuterated jasmonic acid (D-JA), deuterated zeatin (D-zeatin), and deuterated salicylic acid (D-SA) (Sigma–Aldrich, St. Louis, MO, USA) were also used. For hormone measurements, a chromatographic column (C18 QuECherS packing) (Amperex, Shanghai, China) along with chromatographic-grade methanol and acetonitrile (Sigma–Aldrich, St. Louis, MO, USA) were used. The detailed gradient parameters for HPLC (Table S2), mass spectrum parameters (Table S3), and selected monitoring indicators for protonation or deprotonation reactions of phytohormones (Table S4) are displayed in the Supplementary Data. Mobile phase: (methanol/0.1% formic acid): (water/0.1% formic acid).

### 4.8. cDNA Extraction and qRNA Analysis

Rice samples subjected to different Se treatments (0 and 80 μM) were collected, and RNA was extracted from rice roots, stems, and leaves under Se toxicity stress using an RNA extraction kit (19291ES50, 50 T, Yeasen Biotechnology, Shanghai, China). A reverse transcription kit (11119ES60, 100 T, YEASEN, Shanghai, China) was used to obtain cDNA. A 20 μL reverse transcription system (5 μL of RNA, 7 μL of RNase-free H_2_O, 3 μL of 5 × gDNA digester mix, and 5 μL of 4 × Hifair III Super Mix plus) was prepared under the reaction conditions (25 °C for 5 min, followed by reaction at 55 °C for 15 min, and 85 °C for 5 min) [112]. qRT–PCR was implemented using a real-time fluorescent quantitative PCR assay system manufactured by Bio-Rad (Hercules, CA, USA). The PCR system consisted of 20 μL, including 8 μL of ddH_2_O, 10 μL of Universal Blue qPCR SYBR Green Master Mix (11184ES08, YEASEN, Shanghai, China), 0.5 μL of reverse primer, 0.5 μL of forward primer, and 1 μL of cDNA. The PCR conditions were as follows: 95 °C for 30 s, followed by 95 °C for 5 s, 58 °C for 60 s, and 72 °C for 30 s. This process was repeated for 45 cycles, and the final reaction was preserved at 16 °C for 20 min [124]. The 2^−ΔΔCT^ method, in which *OsActin* (*Os03g0718100*) is used as the internal parameter, was used for data analysis [125]. The primers applied to qPCR in this study are exhibited in Table S5.

### 4.9. Data Processing

All the data were analyzed using Microsoft Excel 2010 (Microsoft, Redmond, WA, USA) and SPSS version 19.0 software (IBM Corporation, New York, NY, USA). The data were analyzed for significant differences via Duncan’s multiple comparison test or the *t*-test [112].

## 5. Conclusions

This study investigated the physiological regulatory mechanism of selenium stress in rice. Initially, an increase in selenium levels was observed in rice cells due to the presence of a large amount of exogenous selenium during selenium poisoning stress. This increase in selenium activated a cellular defense mechanism in rice, leading to changes in the levels of MDA, APX, CAT, POD, SOD, proline, soluble sugar, and soluble protein in rice. As a result, the rice cells were protected, and oxidative damage was reduced. Furthermore, under selenium toxicity stress, selenium ions might influence the expression levels of genes related to selenium metabolic pathways and metal ion transportation. These inductions resulted in variations in the expression of SMT-related genes (*SMT1*, *SMT2*, and *SMT3*) and metal ion transport genes (*Nramp3* and *Nramp5*), leading to changes in plant hormone levels (IAA, Zeatin, SA, JA, and ABA). Finally, these changes led to symptoms such as a decrease in biomass, growth restriction, and an unnormal aggregation of metallic ions. The findings of this research lay a basis for further study on the molecular and physiological mechanisms of rice’s response to selenium stress. Additionally, these results will provide an academic basis for the cultivation of new Se-rich rice varieties through breeding efforts.

## Data Availability

Data is contained within the article.

## Supplementary Materials

The following supporting information can be downloaded at https://www.mdpi.com/article/10.3390/plants13111580/s1: Table S1: Composition and content in hydroponic solution; Table S2: The detailed gradient parameters for HPLC; Table S3: mass spectrum parameters; Table S4: the selected monitoring indicators for protonation or deprotonation reactions of phytohormones; Table S5: the primers applied to qPCR in this study; Figure S1: The retention time and HPLC chromatograms of Zeatin, SA, JA, IAA, and ABA. The retention time and HPLC chromatograms of Zeatin, SA, JA, IAA, and ABA were displayed orderly from top to bottom.
