# Supplementary material for: High Concentrations of Se Inhibited the Growth of Rice Seedlings
Source: Plants (Basel). 2024 Jun 6;13(11):1580. doi: 10.3390/plants13111580 (PMC11174541; doi:10.3390/plants13111580)
Supplement: Supplementary file 1 [file plants-13-01580-s001.zip › plants-3038798-supplementary-6.4/Supplementary Materials-2024.6.4/Table S2-S4 and Figure S1.pdf]

Table S2. Gradient parameters of HPLC

Time (min)	Flow velocity (mL/min)	A%
0-1	0.3	20
1-3	0.3	Increasing from 20 to 50
3-9	0.3	Increasing from 50 to 80
9-10.5	0.3	80
10.5-10.6	0.3	Decreasing from 80 to 20
10.6-13.5	0.3	20

A means methanol containing 0.1% formic acid.

Table S3. Mass spectrum parameters

Parameter type	Value or category
Ionization mode	ESI positive and negative ion mode
Scan type	Multiple reaction monitoring (MRM)
Curtain gas	15 psi
Spray voltage	+4500 v, -4000 V
Atomizing gas pressure	65 psi
Auxiliary gas pressure	70 psi
Atomization temperature	400 ℃

Table S4. Selected reaction monitoring conditions for protonated or deprotonated plant hormones ($[M+H]^+$ or $[M-H]^-$)

Hormones	Polarity	Parent ion （m/z）	Daughter ion （m/z）	Decoupling voltage (V)	Collision energy (V)
IAA	+	176.1	129.8*/102.9	65	12/42
ABA	-	263.1	153.1*/204.2	-60	-14/-27
Zeatin	+	220.3	147.9*/202.1	92	22/16
SA	-	137	92.9*/65	-50	-20/-39
JA	-	209.2	59.1*	-54	-16

Note: Those marked with "*" are quantitative ions.

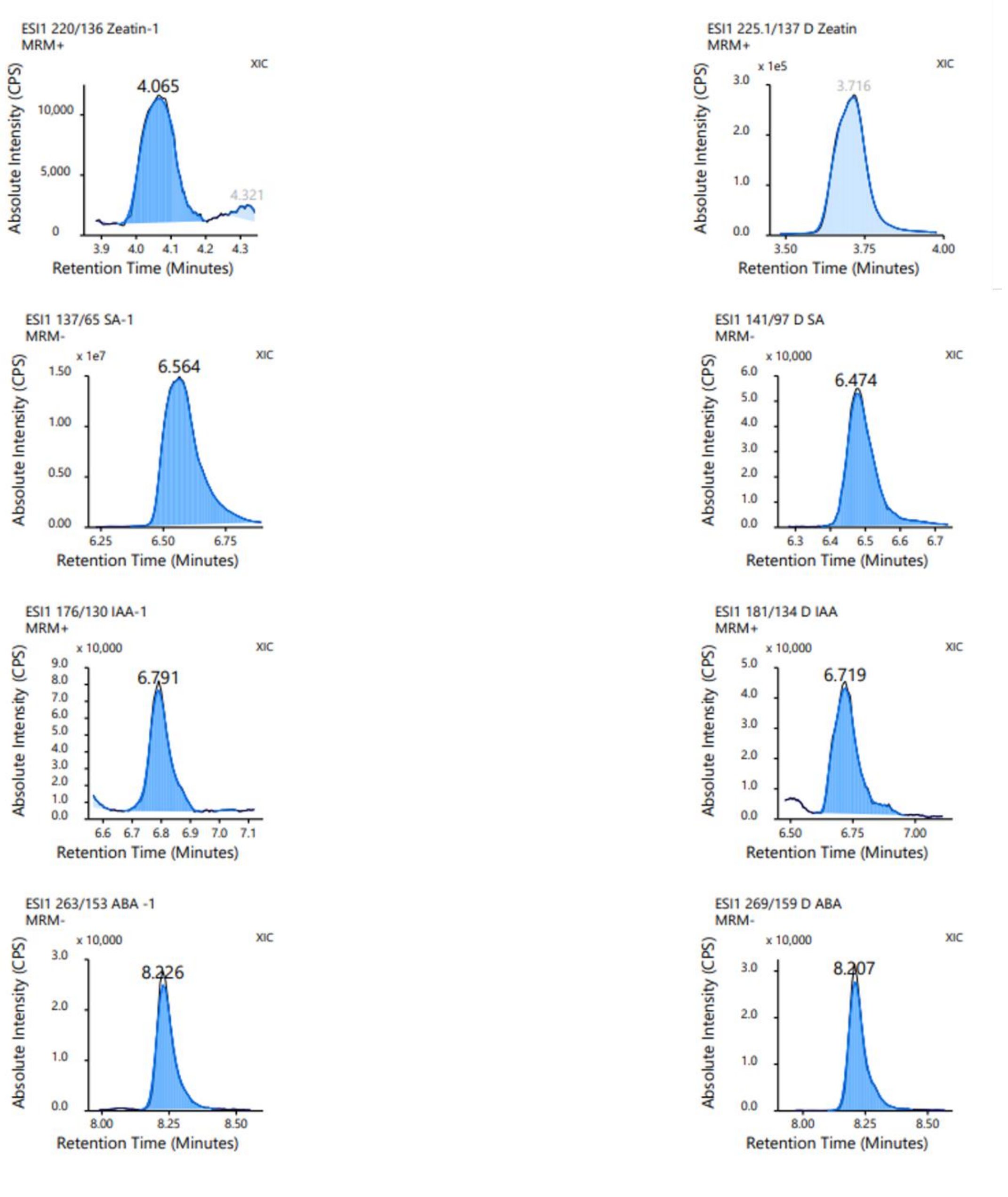

Figure S1. The retention time and HPLC chromatograms of Zeatin, SA, JA, IAA, and ABA. The retention time and HPLC chromatograms of Zeatin, SA, JA, IAA, and ABA were displayed orderly from top to bottom.
